# Dental Education

**Published:** 1869-04

**Authors:** Richard B. Winder


### ARTICLE IV.

### *Dental Education.*

By Richard B. Winder, D.D.S.

Dentistry has always impressed me as one of the most important, and necessary specialties of Medical Science, but to attain any high degree of proficiency in it requires so much careful investigation, observation, and severe application, that it may indeed be reckoned among the Herculean tasks.

The veriest Tyro in the professsion will at once admit the necessity of a complete and thorough understanding of the teeth, or organs of mastication. This much knowledge is absolutely essential, and its attainment necessarily invokes a rigid enquiry into the origin, formation, size, appearance, situation, composition, functional characteristics, diseases, treatment &c., of these organs. How can we acquire this indispensable information? Is there any Royal road leading to it, which we can instinctively follow, and by which, without exertion, we can gain our end? The answer comes at once, No! It can only be reached through the most thorough teachings of those branches of science which treat of these things, and from their proper application through actual experience and constant practice.

But can we stop here? No! A knowledge of the teeth alone is not sufficient; they are so intimately connected with other surrounding and contiguous organs that we must perforce gain also an understanding of these second organs, and their surrounding and contiguous organs; and the only means of acquiring such information is by giving all these parts the same careful study and strict attention. So that, when we once find ourselves within the labyrinth of the mysteries of organic life, its vital phenomena, functional characteristics, structure, diseases, treatment, &c., we have but one means of escape—one mode of relief, and this consists in a thorough knowledge of the whole animal economy; and we must understand it, both in its normal, and abnormal conditions, else we could not detect those aberrations from a healthy organism, which are known as disease; this accomplished, we must next turn our attention to, and learn those remedial agents which induce a restoration of the normal condition, and we must also learn *their* proper application. I again ask—How can this knowledge be attained? and am free to confess that I know of no other means, except through a careful investigation, and appreciation of the truths taught us in Anatomy, Physiology, Chemistry, Pathology, Etiology, Therapeutics and Treatment, or the the Principles and Practice of Dental Surgery, and Mechanical Dentistry. These then are the departments or branches of science, from which we consider the absolute essentials of proficiency in our profession are to be gained.

The question now arises—What are the best advantages we can avail ourselves of in acquiring these essentials? I unhesitatingly answer, through the medium of well conducted and well regulated Dental Schools; we there find each special branch, or at least so much of it as has any practical bearing upon the profession, taught separately, and thoroughly demonstrated, and by a class of men who are most eminently qualified for this purpose. Surely then, if a student will properly devote his time and attention, he must succeed in laying a proper foundation for future proficiency and preferment,whether he attains such degree of excellence in after life, depends upon his own exertions, if he continues his work so thoroughly begun, and goes on improving his time in still further careful enquiry, and investigation, and gives it his constant care, and attention, his success is certain, he will become an honor to his profession and will reach the very acme of his highest hopes, but he will never cease to feel the greatest affection for his Alma Mater, and the dear old Professors, who labored so faithfully to teach him those essentials, which properly improved, and added to, have since induced his present high standing. We have then no means of success in gaining proper information except through these schools. The days of Dentistry as a mere trade, in the hands of ignorant blacksmiths and barbers, has gone by forever. The profession is now reckoned among the Surgical Specialities of Medicine, has been so acknowledged, and is therefore placed upon an equal footing with them. It consequently demands the same degree of attention and scientific attainments; It can no longer be taught satisfactorily either to the student, or the patrons of the profession, in the operating room and laboratory of some pretending Dentist. There are but two classes of men who would take issue with me on this question, to the first belong those, who are so ignorant themselves, that they cannot appreciate or tolerate learning in others, they are like common sailors on board ship, who always have the greatest contempt for educated officers who have been taught seamanship thoroughly and scientifically, while they never fail to express the highest admiration for him, who has crawled into the "horse pipes" of the ship forward, and blunderingly worked his way aft. The second class are those persons, who, having arrived at some degree of proficiency in the profession themselves, are ever ranting against our Dental Colleges, and complaining that their standard of graduation is too low. They entirely forget, that from a student who has just received his diploma, the world does not expect the same standard of proficiency, which we ought to find in those old practitioners, who, perhaps, in the start, had the same advantage of collegiate education, and since then, the still further advantages of long practice, close observation and continued application. This high degree of excellence is not expected of the graduates of Law, Medical or Theological Schools. Why then demand it from the schools of Dentistry ? I always feel that the opposition, and cavilling of this second class, proceed from either a mean fear of honorable competition which might prove deleterious to their own interests, or from a tyrannical desire to keep under foot every aspirant for professional favor, who has not, like themselves or other tradesmen, been bound in apprenticeship, and served out their full time ; but I am wandering from my subject. The system of preparation at our Dental Colleges is without question equal, if not superior to any of the courses of instruction in other professional schools, and in the conferring of diplomas they are equally rigid, whether we regard the requirements of time occupied in preparation, or the amount of knowledge to be possessed by each candidate for graduation ; and please recollect, so far as Medicine is concerned, that while it takes but two courses of instruction at these schools to teach *everything* pertaining to the whole science of Medicine, our Dental Schools devote the same length of time to one single specialty of this general science. Which ought to be, and which is the best prepared ? he who has applied himself two years in acquiring all the various branches of general Medicine, or he who has devoted the same time and attention to one single specialty ?

We have spoken of the essentials of proficiency in Dentistry, the requirements of the profession, however, go further and beyond these, and " ex rei necessitate " inexorably demand the closest scrutiny and most accurate knowledge of Physiognomy, and those laws of undeviating symmetry in each distinctive part, which taken as a whole, give so much exquisite beauty to man. In using the term Physiognomy, I have considered it in its etymological sense, a knowledge of nature, but have employed it specially, to designate the proper configuration and expression of the countenance. The human face, involuntarily reflecting as it does all the varied emotions of the mind, has been called the "mirror of the Soul" and has ever been esteemed the perfection of the Almighty's handiwork, and his most beautiful of all created things. How necessary then in the profession of Dentistry, that we should thoroughly understand those exact laws, and their relations to proportion, and delineation, which the distinctive parts of the mouth (that most expressive feature of the face) require in forming such perfection of expressive beauty? How necessary that each separate organ should be thoroughly understood, and its co-workings in the general harmony appreciated? How necessary that we should be able to detect the slightest aberration from Nature's laws regulating these exquisitely beautiful formations? How necessary such a knowledge in our attempts at artificial replacement of teeth? distinctive organs, which have so much to do with the proper formation of the mouth and its functional beauties? and how easily, in the absence of such knowledge, we might disregard all law of symmetry, and produce distortion and even hideousness.

There is no feature of the face, which has such an absolute control over the various emotional expressions as the mouth, this is necessarily so and for an obvious reason;—by far the largest number of the muscles of the face and neck, denominated "par excellence, the muscles of expression," have their origin or insertion in the superior and inferior maxillary bones, and are consequently almost entirely dependent upon these organs in the faithful execution of their functional duties. The slightest alteration in the antagonizing proximity of the jaws would necessarily produce a corresponding change in these muscular relations, and the least aberrating movement from Nature's own perfection of position, and symmetrical movement, would necessarily disarrange and destroy those exquisite proportions which constitute that beautiful kaleidoscope of the soul, the human face. Ecclesiasticus observed, "that the heart of a man changes his countenance, whether it be for good or evil, and a merry heart makes a cheerful countenance," deprived then of the influence of the laws regulating this nonpareil of physical beauty, the face would no longer reflect a truthful portraiture of the internal emotions, and would thus lose its most valued and fascinating attractiveness. The dimpled cheek of fulsome beauty would at once disappear; the earnestness and pathos of impassioned feeling could not be exhibited, or at all understood; the tender, trusting look of requited and confiding love would never again speak it's volumes of encouragement, and promise of bright hopes yet to be realized; a mother's devotion would no longer be depicted in it's accustomed instinctive and impressive characteristics; the stern expression of fixed determination would lose its power, and the human face with all its beauties would be lost; we could not read it, the movements of the varying muscles would be expressional hieroglyphics, which could not be deciphered; a ghastly grin might be the sad exchange for what was once a most radiant and joy-beaming smile; all the features might become more or less involved in these horrible metamorphoses, and the countenance, that most beautiful master work of the Creator utterly destroyed. Nature is as as unchanging in her laws of proportions and motional relations, as she is in other matters, and any infringement upon these would carry with it its own unmistakable evidence of aberration, and its consequent deformity. Take the fractional parts, artistically spoken of as the "curved lines of beauty," which as a whole, make up the contour of the face of the celebrated statue of Apollo Belvidere, our beau ideal of manly beauty, or take the face of the equally well known statue of Venus de Medicis, our highest conception of lovely woman, and see what a very slight alteration of feature it would require, to entirely destroy the harmony of beauty in these much admired works of Art, and render them unattractive, or even repulsive? How carefully has the sculptor studied the configuration of nature, and in the proper conception of them in their true relative proportions, and delineations, he has produced, by mechanical skill, works of merit, which in their faultless reproduction, have given us the same exquisite beauty of expression and contour which belonged to the original model. How necessary then a knowledge of Physiognomy must be to a proper attainment of professional proficiency, whether it serves us in the operative surgery of Dentistry, or in it's mechanical department, the artificial replacement of teeth? It is like the Sculptor's last inspired touch to his block of marble, or the painter's last tint to his finished picture. It is the study,—It is the perfection of nature realized. It teaches and enables us in our practice to correct deformity and mitigate and modify repulsiveness, and it restrains us from improperly interfering with natural expression and configuration. An accurate knowledge therefore of those laws regulating physical beauty and a skilfulness of execution in carrying out their inexorable demands, should be ever a most desirable attainment with him, who would give Dentistry an honorable, reputable and enviable standard among the professions.

